# Role of Point-of-Care Ultrasound in Inpatient Perioperative Medical Management: A Systematic Review

**DOI:** 10.3390/jcm14207429

**Published:** 2025-10-21

**Authors:** Dhairya M. Jarsania, Mike J. Breunig, Gururaj J. Kolar, Meltiady Issa, Ryan Kingsley, Mohammed Nadir Bhuiyan, Cynthia J. Chelf, Robert W. Kirchoff

**Affiliations:** 1 Division of Hospital Internal Medicine, Mayo Clinic, Rochester, MN 55905, USA; breunig.michael@mayo.edu (M.J.B.); kolar.gururaj@mayo.edu (G.J.K.); issa.meltiady@mayo.edu (M.I.); kingsley.ryan@mayo.edu (R.K.); 2 Division of General Internal Medicine, Mayo Clinic, Rochester, MN 55905, USA; bhuiyan.mohammed@mayo.edu; 3 Department of Library-Public Services, Mayo Clinic, Rochester, MN 55905, USA; chelf.cynthia@mayo.edu; 4 Division of Hospital Internal Medicine, Mayo Clinic, Phoenix, AZ 85054, USA; kirchoff.robert@mayo.edu

**Keywords:** POCUS, point-of-care ultrasound, perioperative, preoperative, postoperative

## Abstract

**Background:** Point-of-care ultrasonography (POCUS) is becoming an increasingly relevant tool in hospital medicine, but its effective application in inpatient perioperative medicine remains to be determined. Much of the POCUS literature describes its use by anesthesiologists to evaluate cardiac function, volume status, pulmonary findings, and gastric volume. **Objective:** To identify, evaluate, and synthesize all available literature investigating the use of point-of-care ultrasound and associated clinical outcomes in inpatient perioperative medical management. **Patients and Methods:** A systematic review was designed using the PRISMA guidelines with sources of literature including Ovid, PubMed, Scopus, and the Web of Science. Literature search was conducted for published works between 1 January 2002 to 8 February 2024. **Results:** Three hundred sixty-seven abstracts were identified in our search, and, ultimately, 24 studies were included in this review. Most studies were done by anesthesiology evaluating cardiopulmonary and gastric POCUS. Studies supported using POCUS to expedite cardiac examination, promptly diagnose postoperative pulmonary complications, and optimize surgical timing. **Conclusions:** POCUS is a versatile tool in the perioperative setting; however, few studies were powered to assess clinical outcomes, and even fewer showed conclusive evidence of improved clinical outcomes. Furthermore, only two studies evaluated the use of POCUS specifically by acute care providers; more extensive studies are needed from their perspective as they take on increasing perioperative responsibilities.

## 1. Introduction

Point-of-care ultrasonography (POCUS) has emerged as a popular tool in hospital medicine due to its portability, ease of use, and ability to provide real-time, dynamic imaging at the patient’s bedside [1]. Its versatility has revolutionized the diagnostic process, offering rapid and non-invasive insights that aid in timely decision-making [2]. Typical applications of POCUS include assessing cardiac function, identifying fluid collections, and guiding procedures such as central line placement and thoracentesis. Additionally, POCUS plays a pivotal role in diagnosing pathologies such as systolic heart failure, pericardial tamponade, pleural effusions and consolidations, venous thromboembolisms, and abdominal aortic aneurysms [3]. POCUS for bedside diagnosis has been well studied with many RCTs and clinical guidelines supporting its use [4,5,6]. POCUS certifications are available through multiple medical societies, including the Society of Hospital Medicine, the American College of Physicians, the American College of Chest Physicians, the Alliance for Physician Certification and Advancement, and the American Academy of Physician Assistants [7,8,9,10,11].

Multiple specialty societies, such as the American Society of Echocardiography, American College of Emergency Physicians, and European Society of Anesthesiology, offer suggestions for the use of POCUS in perioperative cardiac function assessment, identification of fluid status, and regional anesthesia guidance [4,12,13]. These serve as a crucial framework for harnessing the full potential of POCUS in perioperative medicine. While POCUS adoption has increased significantly amongst anesthesiologists, a direct correlation between perioperative decisions guided by POCUS and demonstrable changes in patient outcomes remains largely unestablished. Further research is needed to quantify the impact of POCUS-informed decisions in this setting. This systematic review attempts to synthesize the evidence regarding the impact of POCUS on clinical outcomes in the inpatient perioperative setting to help guide clinicians in the medical management of perioperative patients.

## 2. Methods

**Figure 1:**
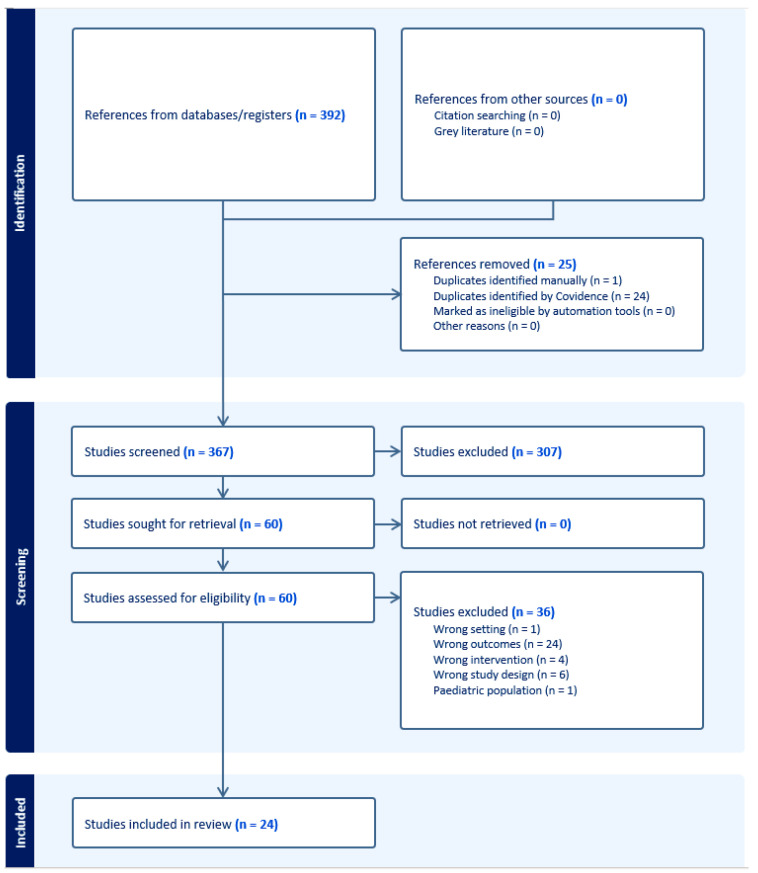
PRISMA diagram for systematic review.

This study utilized a systematic review design performed in compliance with the Preferred Reporting in Systematic Reviews and Meta-Analyses (PRISMA) guidelines [14,15]. Literature search of publications within the past two decades was conducted with the assistance of a medical librarian on 8 February 2024. Sources included Ovid (Embase & Cochrane), PubMed (MEDLINE), Scopus (Elsevier), and Web of Science with query terms including point-of-care, ultrasound, POCUS, adult, and perioperative. Inclusion criteria included adult patients, planned/performed surgical intervention, use of POCUS during evaluation, and studies with measured clinical outcomes. For this study, clinical outcomes were broadly defined as patient and clinician-reported outcomes, including but not limited to changes in symptoms, clinical status, mortality, comorbidities, and hospital metrics. Studies were excluded if they involved only pediatric patients, outpatient POCUS evaluation, intra-operative use of POCUS, or were non-peer-reviewed publications, letters to the editor, case reports, or in a non-English language (Figure 1). Each abstract, full text, data abstraction, and bias assessment was completed by two different reviewers, with a third reviewer as arbitrator. Bias assessment for the non-randomized cohort and case-control studies included was accomplished using the Newcastle–Ottawa grading scale, with 1 point for each star on the cohort studies scale for a maximum of 9 points. Studies totaling 7–9 points were graded as “good,” 4–6 points as “moderate,” and <4 points as “poor” [16]. Randomized control studies (RCTs) were assessed for bias using the Cochrane Risk of Bias 2.0 tool [17]. Operator ultrasound experience was determined to be “expert” if there was mention of certification in ultrasonography, “experienced” if there was mention of the operator being experienced or formally trained in US, “limited training” if there was specific mention of limited or brief training in US, and “unknown” if there was no mention of US experience or level of experience was unclear. The web-based Covidence platform was used to screen the literature and extract data, and integrated functions were used to determine proportionate agreement [18]. Proportionate agreement was calculated by dividing the total number of agreements by the total number of items for the respective screening stages. A meta-analysis was not conducted due to the significant variation in study methods, including patient populations, interventions, including different types of POCUS, and the different primary and secondary outcomes studied within the limited number of articles that were included in the review.

## 3. Results

Our literature search strategy yielded 367 abstracts, of which 60 met the inclusion criteria (Supplementary Materials). After excluding articles for lack of clinical outcomes (n = 24), incorrect study design (n = 6), lack of POCUS as an intervention (n = 4), outpatient setting (n = 1), and pediatric population (n = 1), 24 studies were included in the review [19,20,21,22,23,24,25,26,27,28,29,30,31,32,33,34,35,36,37,38,39,40,41,42]. Most of the studies were cohort or case-control studies except for Szabo (2023), Ravetti (2023), and Cavallari (2015) [25,37,39], which were RCTs. Five reviewers screened titles and abstracts with a proportionate agreement equal to 84%, and seven reviewers screened full texts with a proportionate agreement equal to 82%. Based on the Newcastle–Ottawa grading scale, all studies were of “good” quality except for Cowie (2011) [27], which was of “moderate” quality (Supplementary Materials). The intention of utilizing a numerical system was to tabulate the authors’ bias evaluation for ease of presentation, and it is not intended to compare studies to one another. The authors’ general assessment of bias in the studies mimics the quality of the study (from a bias perspective) represented by the tabulated results from using the Newcastle–Ottawa scale. All studies, except for Cowie (2011) [27], had low bias based on the authors’ critical assessment.

The organ systems studied include cardiac (8), pulmonary (7), gastrointestinal (4), vascular (4), renal (3), musculoskeletal (1), and genitourinary (1) in the perioperative setting of various surgeries, with all but 2 being non-cardiac, and orthopedic surgery being the most common of the non-cardiac surgeries. Five studies showed some evidence of impact on clinical outcomes, four showed no impact on clinical outcomes, five studies showed an impact on diagnostic accuracy, five studies showed an impact on anesthetic plan or intraoperative management, and six studies showed an association between US findings and clinical parameters without changes to clinical outcomes. Baseline patient characteristics for each study are listed in Table 1. Sample sizes ranged from 20 to 512 patients, with an average of 112 patients. 12 studies had fewer than 100 patients, and 12 had 100 patients or greater. The average age ranged from 33 to 82 years, with a mean of 62 years, the percent male ranged from 34 to 93, with a mean of 54, and the average body mass index (BMI) ranged from 23.4 to 28.6, with a mean of 25.8. American Society of Anesthesiologists (ASA) classification information was presented in 13 out of the 24 studies, with class 2 being the most common.

A summary of study methods for each study is listed in Table 2. Most studies were done in the United States (5), followed by Canada (3) and Italy (3). The ultrasound operator specialties across the 24 studies were anesthesia (15), cardiology (2), surgery (1), medicine/critical care (2), nursing (1), and not specified in 4 studies. Most of these operators were “experienced” with POCUS, with a few being “experts” or having “limited training”. Ultrasound devices varied greatly between studies, but nearly all devices were either cart-based or laptop design, except for devices used in Ramsingh (2021), Cavallari (2015), and Cutright (2011), which were hand-held [25,29,36]. Chui (2023) [26] did not have a listed device.

The specific aims, measurements, and conclusions of each study varied substantially, even when assessing similar organ systems (Table 3). Cardiac ultrasound was utilized to assess baseline cardiac function, presence of a patent foramen ovale, and volume status [19,20,25,27,30]. Lung ultrasound was utilized to assess for atelectasis and pulmonary congestion [21,31,38,41]. Gastric ultrasound was utilized predominantly to assess gastric volume [28,32,34,40]. Vascular and renal ultrasound was utilized to assess volume status and risk for acute kidney injury [33,35,39]. Multisystem evaluations, including the heart, lungs, and/or inferior vena cava, were utilized in four studies to assess volume status [26,36,37,39]. Results regarding conclusions from each study and their implications are considered in the discussion section.

## 4. Discussion

Limited research has been conducted on outcomes-based investigations that employ point-of-care ultrasonography in inpatient perioperative medicine. Notably, the existing literature lacks perspectives from acute care providers. This comprehensive review reveals that the use of POCUS in perioperative medicine derives primarily from the anesthesia standpoint, predominantly concerning its impact on anesthetic plans and post-anesthesia care. The studies within this domain largely revolve around cardiac, pulmonary, and gastric POCUS.

Most of the study patients are classified as American Society of Anesthesiologists (ASA) class 1 or 2. In contrast, inpatient medicine consults for presurgical evaluation generally involve ASA class 3 and 4 patients [43,44]. Furthermore, the ultrasound operators in these studies predominantly hail from anesthesia and cardiology specialties, both of which undergo formal ultrasound and POCUS training during their respective training programs. This specialized training might elevate the significance of their findings compared to studies involving general medicine providers with varied levels of POCUS training. However, there is evidence, including studies beyond the scope of this review, validating the ability of novice POCUS users to accurately assess cardiac function and make common diagnoses after limited training [45,46]. Therefore, cardiac POCUS is a potential starting point for assessing the impact of acute care providers using POCUS in inpatient preoperative evaluation on patient outcomes. The following sections highlight the application of POCUS in inpatient perioperative medical management of the five most studied organ systems.

### 4.1. Cardiac

Andruszkiew (2015) and Cowie (2011) [20,27] demonstrate that even when performed by anesthesiologists with basic training, focused cardiac POCUS can significantly alter perioperative decisions. Additionally, Cowie (2011) and Cavallari (2015) [25,27], an RCT, highlight that focused transthoracic echocardiography (TTE) and hand-held TTE devices, respectively, can provide satisfactory diagnostic quality with the advantage of shorter wait times and expedited exam performance. In contrast, Chui (2023) [26] indicates that while POCUS does not necessarily change anesthetic plans, its use may avert surgical delays and assist in assessing severe cardiopulmonary conditions. These studies suggest cardiac POCUS may be a more practical exam than formal TTE for most perioperative patients.

Furthermore, targeted applications—such as using the velocity–time integral of the left ventricular outflow tract-passive leg raise to predict post-induction hypotension (Aissaoui, 2022) [19] or detecting patent foramen ovales potentially linked to postoperative delirium (Gai, 2018) [30]—underscore the evolving role of POCUS in risk stratification. Lastly, the integration of combined cardiac and lung assessments, as reported by Ramsingh (2021) [36], appears beneficial in reducing post-anesthesia care unit length of stay in vitally unstable patients.

### 4.2. Pulmonary

Basumatary (2023) and Wu (2023) [21,41] highlight that lung ultrasound can identify lung congestion, atelectasis, and aeration before the manifestation of clinical signs and symptoms during the early postoperative period. A more quantitative approach using the lung ultrasound score has proven to be a valuable metric in identifying patients at risk of or in the early phase of postoperative pulmonary complications (Szabó, 2021) and correlates with prolonged weaning time for respiratory support (Goel, 2020) [31,38]. These studies show that point-of-care lung ultrasound is a vital tool in the early detection and management of pulmonary complications in the perioperative setting.

### 4.3. Gastrointestinal

Cozza (2021) [28] demonstrates that a preoperative dilated antrum identified via POCUS is significantly related to adverse postoperative outcomes, suggesting that targeted ultrasound follow-up may be useful in optimizing postoperative nutrition and antiemetic therapy. Van de Putte (2017) [40] finds that patients may present with full stomachs despite adhering to recommended fasting guidelines, which indicates a prime opportunity for the use of POCUS in pre-anesthetic management to mitigate risks. Post-operatively, Lamm (2022) [34] reports that patients with full stomachs on postoperative day one after colorectal surgery experience a delayed recovery of gastrointestinal function, as indicated by prolonged GI3 recovery, and Haskins (2017) [32] observes that intra-abdominal fluid extravasation following hip arthroscopy is correlated with increased postoperative pain, thereby providing predictive insight into a patient’s clinical course. Collectively, these studies underline the critical role of perioperative gastrointestinal evaluation in tailoring management strategies. This could be particularly important for patients on GLP-1 receptor agonists, which can slow gastric emptying and increase the risk of retained gastric contents and aspiration [47].

### 4.4. Vascular/Renal

Studies addressing vascular ultrasound evaluation are largely focused on the correlation between vasculature, fluid status, and renal function. Kaydu (2019) [33] demonstrates no relationship between inferior vena cava parameters and blood-urea-nitrogen/creatinine ratio to predict perioperative dehydration, but Szabo (2023) [39], an RCT, successfully implemented a preoperative, ultrasound-based fluid administration protocol that prevents early intraoperative hypotension and guides timing of fluid administration. Consistent with this, Beaubien-Souligny (2018) [22] shows an association between a decrease in the renal resistance index and an increase in cardiac output following passive leg raise after cardiac surgery.

Several studies looked at the impact of vascular POCUS on the development of AKI. Pettey (2022) and Yamanaka (2022) [35,42] utilize hepatic vein flow wave ratios and renal artery pulsatility index, respectively, to show an association between these parameters and perioperative AKI. Brusasco (2023) [23] refines this association to predict perioperative AKI using intra-renal venous flow patterns. A multivariate model focused on IVC, renal, and hepatic vasculature may have more promising outcomes on AKI prediction and prevention. However, Ravetti (2023) [37], an RCT focused on assessing the impact of bedside lung, IVC, and cardiac ultrasound on hemodynamic management in the immediate post-operative period, did not show benefits in the incidence of post-operative AKI.

Many of the POCUS applications reviewed by the system above may be applicable to inpatient medicine; accordingly, inpatient practice would benefit from studies exploring the use of POCUS specifically by acute care providers. Future studies on perioperative medical management utilizing POCUS should focus on patient outcomes while meticulously defining baseline patient characteristics and clinical measurements in addition to POCUS parameters. Essential patient characteristics for data collection include age, sex distribution, BMI, ASA class, planned surgery, and highest metabolic equivalent level, as well as results of commonly used perioperative risk calculators, including the National Surgical Quality Improvement Project, myocardial infarction-cardiac arrest, revised cardiac risk index, and the ARISCAT score for post-operative pulmonary complications. For methodology, ultrasound operator specialty, operator training, ultrasonography device, ultrasound views, and parameter data, clinical translation of imaging findings (e.g., cardiac function vs. change in left ventricle diameter), and most importantly, clinical outcomes should be considered. These may include length of stay (PACU, ICU, or hospital), morbidity and mortality, diagnostic accuracy, time to diagnosis or intervention, complication rates, and readmission rates. The standardized reporting of baseline data and clinical measurements would improve comparability between studies by allowing researchers and readers to easily compare patient populations, interventions, and outcomes, along with allowing more rigorous review of the literature via meta-analysis. This will facilitate the application of future POCUS research in the clinical setting, propelling the field of perioperative medicine forward.

Limitations of this systematic review include the heterogeneity of the studies included, which makes it challenging to synthesize results and draw meaningful conclusions. The studies vary in design and methods, operator experience or training, patient characteristics, consistency of findings (most studies focus on cardiopulmonary POCUS and evaluation), and clinical relevance in terms of the studied patient outcomes. Inherent to their study design, the three RCTs provide the most robust evidence on comparing formal echocardiography with cardiac POCUS and managing intraoperative fluid administration. Given the multitude of different methods and outcomes studied amongst the included studies, no subgroup analysis was conducted. Broad variations in patient demographics may affect the generalizability of the findings; thus, findings from each study should be taken in the context of their respective patient population. The review is also susceptible to publication and reporting biases, given the tendency for positive studies to be published more frequently than those with negative or inconclusive results. Small sample sizes for some studies may have led to imprecise estimation of the effect. In addition, direct application of these studies on inpatient management will likely vary based on the practice setting, available resources, and staff experience at each institution. Limitations in this review’s methods include a lack of registration with PROSPERO, an international systematic review registry, and intentional exclusion of grey literature. These limitations in methods are thought to have a negligible impact on the results and implications of this review. Grey literature is generally not peer-reviewed and thus introduces additional bias and inconsistency within systematic reviews, so the authors felt that inclusion of grey literature would negatively impact the quality of the study while providing limited information on patient outcomes. However, grey literature can help reduce publication bias and provide unique or more up-to-date information, so its exclusion is a noted limitation. Lack of PROSPERO registration does restrict the review’s openness and reproducibility, particularly while a study is in progress; however, a thorough literature search was completed with the assistance of a medical librarian to ensure that similar reviews were not previously published, and we believe the transparent methodology, including the search strategy included in Supplementary Materials, provides the resources for reproducibility if needed.

## 5. Conclusions

This review demonstrates the versatility of POCUS in perioperative management. Cardiopulmonary and gastric POCUS imaging have the most data to guide management for post-operative ICU care and appropriate surgical timing, respectively. Very few studies were powered to assess clinical outcomes, and even fewer showed overlapping evidence to strongly suggest improvement in clinical outcomes secondary to the use of perioperative POCUS. Additionally, more extensive studies are needed to truly evaluate the benefits or harms of using POCUS in inpatient perioperative medical management, especially from the acute care provider perspective, as they undertake more perioperative responsibilities. Future studies should focus on clinical questions answerable with POCUS for preoperative medical evaluation and non-ICU post-operative care in the less-controlled environment of inpatient wards, with an emphasis on evaluating associated clinical outcomes.

## Figures and Tables

**Table 1:** Baseline patient characteristics. BMI (body mass index), ASA (American Society of Anesthesiology), n/a (not available).

Study ID	Total Sample Size	Age (Average)	Sex (% Male)	BMI (Average)	ASA Class (Mode)
Aissaoui 2022 [19]	64	62	56	25.5	2
Andruszkiewicz 2015 [20]	155	57	46	26.2	2
Basumatary 2023 [21]	70	38	n/a	23.4	<3
Beaubien-Souligny 2018 [22]	30	68	n/a	28.6	n/a
Brusasco 2023 [23]	173	68	91	25.0	2
Canales 2022 [24]	32	62	34	27.6	3
Cavallari 2015 [25]	100	71	48	n/a	n/a
Chui 2023 [26]	196	82	37	26.0	3
Cowie 2011 [27]	170	n/a	51	n/a	n/a
Cozza 2021 [28]	41	62	68	24.1	n/a
Cutright 2011 [29]	47	62	43	n/a	n/a
Gai 2018 [30]	202	64	41	n/a	n/a
Goel 2020 [31]	28	53	93	24.6	2
Haskins 2017 [32]	100	33	47	24.2	1
Kaydu 2019 [33]	30	80	30	27.1	3
Lamm 2022 [34]	20	60	45	26.2	n/a
Pettey 2022 [35]	152	68	72	n/a	n/a
Ramsingh 2021 [36]	128	65	55	26.2	3
Ravetti 2023 [37]	111	56	48	n/a	2
Szabó 2021 [38]	67	67	55	26.5	2
Szabo 2023 [39]	76	65	54	27.2	2
Van de Putte 2017 [40]	512	48	55	25.8	n/a
Wu 2023 [41]	93	56	58	24.1	n/a
Yamanaka 2022 [42]	100	69	58	n/a	n/a

**Table 2:** Summary of methods. Abbreviations: GE (General Electric), USA (United States of America), ENT (ear, nose, throat), PA (pulmonary artery), CT (computed topography), TTE (transthoracic echocardiogram), mmHg (millimeters of mercury), PACU (post-anesthesia care unit).

Study ID	Country	Operator Specialty	Study Dates	Ultrasound Device	Operator US Experience	Organ System of Interest	Type of Surgery	Population Description
Aissaoui 2022 [19]	Morocco	Anesthesia	Feb–May, 2020	LOGIQ e Machine (GE Healthcare, Oslo, Norway)	Experienced	Cardiac	Abdominal	Patients older than 50 years scheduled for elective abdominal surgery
Andruszkiewicz 2015 [20]	Poland	Anesthesia	Oct–Dec, 2014	Sparq System (Philips Ultrasound, Bothell, WA, USA)	Experienced	Cardiac	ENT; General; Neurologic; Vascular	Patients older than 18 years scheduled for elective operations
Basumatary 2023 [21]	India	Anesthesia	Dec, 2019–Dec, 2020	SonoSite Edge II (Fujifilm Sonosite, Bothell, WA, USA)	Unknown	Pulmonary	Extrathoracic	Adults undergoing elective surgery seen for pre-anesthesia check-up
Beaubien-Souligny 2018 [22]	Canada	Medicine/Critical care	n/a	CX50 Ultrasound System (Philips Healthcare, Amsterdam, The Netherland)	Experienced	Renal	Cardiac	Patients older than 18 years undergoing cardiac surgery for which a PA catheter would be installed
Brusasco 2023 [23]	Italy	Not specified	Dec, 2019–Apr, 2022	Mindray TE7 (Shenzhen Mindray Bio-Medical Electronics Co., Shenzhen, China)	Expert	Renal	Urologic	Adults patients admitted for major laparoscopic surgery to urology department
Canales 2022 [24]	United States	Anesthesia	Feb, 2019–Mar, 2020	Vivid S6 Ultrasound System (GE Healthcare, Wauwatosa, WI, USA)	Expert	Musculoskeletal	Abdominal; Gynecologic Oncology; Orthopedic/Spine; Urologic Oncology; Vascular	Adult patients scheduled for surgery who had a CT abdomen/pelvis within 90 days of their preoperative clinic visit
Cavallari 2015 [25]	Italy	Cardiology	n/a	Opti-Go Hand-Held (Philips Medical Systems, Andover, MA, USA); iE33 (Philips Medical Systems, Bothell, WA, USA)	Experienced	Cardiac	Non-cardiac	Adult patients referred to cardiac pre-operative evaluation
Chui 2023 [26]	Canada	Anesthesia	May, 2018–Nov, 2021	n/a	Expert	Cardiac and pulmonary	Orthopedic	Adults scheduled to undergo urgent hip fracture surgery
Cowie 2011 [27]	Australia	Anesthesia	May, 2007–Apr, 2010	Acuson Cypress (Siemens Healthcare, Mountain View, CA, USA); Vivid I (GE Medical Systems, Milwaukee, WI, USA); M-Turbo (Sonosite, Bothell, WA, USA); iE33 (Philips Medical Systems, Andover, MA, USA)	Experienced	Cardiac	Non-cardiac	Patients referred to perioperative TTE service by primary anesthetist in surgical case based on set clinical indications
Cozza 2021 [28]	Italy	Emergency Surgery and Trauma	Jan–Jun, 2019	MyLab Gamma (Esaote, Fishers, IN, USA)	Unknown	Gastrointestinal (gastric)	Abdominal	Patients admitted to division of Emergency Surgery and Trauma undergoing urgent abdominal surgery
Cutright 2011 [29]	United States	Nursing	May–Jun, 2009	BladderScan BVI 9400 Hand-Held (Verathon Medical, Bothell, WA, USA)	Limited training	Genitourinary	General; Orthopedic	Adult patients admitted to medical-surgical inpatient unit that met facility criteria for use of bladder scanner per hospital policy
Gai 2018 [30]	Canada	Anesthesia	Mar, 2015–Jun, 2016	Sparq System (Philips Ultrasound, Markam, ON, USA)	Experienced	Cardiac	Orthopedic	Patients older than 18 years scheduled in pre-admission clinic with an appointment for elective primary hip or knee replacement procedure
Goel 2020 [31]	India	Anesthesia	May–Nov, 2017	Sonosite (GE Healthcare, Bothell, WA, USA)	Experienced	Pulmonary	Head and Neck Cancer Reconstruction	Patients aged 18–65 years scheduled for elective head and neck cancer resection surgery followed by free flap reconstruction
Haskins 2017 [32]	United States	Anesthesia	Apr–Mar, 2015	LOGIQ e Ultrasound (GE Healthcare, Wauwatosa, WI, USA)	Expert	Gastrointestinal (non-gastric)	Orthopedic	Patients aged 18–80 years scheduled for outpatient hip arthroplasty
Kaydu 2019 [33]	Turkey	Anesthesia	n/a	SonoSite M-Turbo (Fujifilm, Bothell, WA, USA)	Experienced	Vascular	Orthopedic	Patients older than 60 years with proximal femoral fractures undergoing hip fracture surgery
Lamm 2022 [34]	United States	Not specified	Feb–Jun, 2021	LOGIQ e Ultrasound (GE Healthcare, Boston, MA, USA)	Experienced	Gastrointestinal (gastric)	Gastrointestinal	Patients older than 18 years scheduled for non-emergent colorectal surgery
Pettey 2022 [35]	South Africa and Denmark	Cardiology and Anesthesia	Aug, 2019–Jan, 2020	S6 or S70 Ultrasound System (GE Healthcare, Brondby, Denmark); CXC50 (Philips, Johannesburg, South Africa)	Unknown	Vascular	Cardiac	Patients older than 18 years presenting for cardiac surgery
Ramsingh 2021 [36]	United States	Anesthesia	Aug, 2018–Aug, 2019	Laptop based devices: (Fujifilm Sonosite, Bothell, WA, USA); (General Electric, Boston, MA, USA); Hand-held devices: (Butterfly Network, Guilford, CT, USA)	Limited training	Cardiac and pulmonary	Head and Neck; Thoracic; Abdominal; Urologic; Gynecologic/Obstetric; Orthopedic; Vascular; Other	Adult patients who experienced a mean arterial pressure < 60 mmHG and/or an oxygen saturation < 88% in the PACU
Ravetti 2023 [37]	Brazil	Internal Medicine	Feb, 2018–Mar, 2019	Terason 3000 ultrasound (Burlington, MA, USA)	Expert	Vascular, pulmonary, and cardiac	Neurologic; Hepatobiliary	Adults in the postoperative period of high-risk surgeries admitted to the ICU
Szabó 2021 [38]	Hungary	Anesthesia	Aug, 2019–Jul, 2020	Aloka Noblus (Hitachi Healthcare, Tokyo, Japan)	Experienced	Pulmonary	Abdominal	Patients older than 18 years classified as ASA 2 or 3 scheduled for elective major abdominal surgery
Szabo 2023 [39]	Hungary	Not specified	Dec, 2021–Aug, 2022	Philips InnoSight (Koninklijke Philips NV, Amsterdam, The Netherlands)	Experienced	Vascular	Abdominal	Adults scheduled for elective major abdominal surgery requiring endotracheal intubation.
Van de Putte 2017 [40]	Belgium	Anesthesia	Jan, 2015–Jan, 2016	HD11XE (Philips Healthcare, Andover, MA, USA); LOGIQ e (GE Healthcare, Chicago, IL, USA); SonoSite X-porte (Fujifilm, Bothell, WA, USA)	Experienced	Gastrointestinal (gastric)	Orthopedic; Abdominal; General; Maxillofacial; Gynecologic; Urologic; Endoscopic; other	Patients older than 16 years scheduled for elective surgery requiring general anesthesia
Wu 2023 [41]	China	Anesthesia	Aug–Sep, 2022	Vivid-i ultrasound machine (GE Healthcare, Wauwatosa, WI, USA)	Experienced	Pulmonary	General; Thyroid; Urologic; Gynecologic; Other	Adult patients undergoing elective non-cardiothoracic cancer surgery requiring general anesthesia
Yamanaka 2022 [42]	Japan	Not specified	Mar–July, 2018	ARIETTA60 (Hitachi, Tokyo, Japan)	Experienced	Renal	Gastrointestinal	Consecutive patients undergoing digestive surgery admitted to the hospital

**Table 3:** Summary of objectives and conclusions.

Study ID	Objectives	Organ System of Interest	POCUS Measurement	Conclusion
IMPACT ON CLINICAL OUTCOMES				
Brusasco 2023 [23]	Assess whether perioperative arteriovenous renal blood flow predicts postoperative AKI after major laparoscopic urologic surgery.	Renal	Visualization of renal parenchyma, intra-renal venous flow (IRVF), doppler time-velocity spectra	IRVF pattern predicts perioperative AKI. Combination of biphasic or monophasic venous patterns in conjunction with overt arterial hypotension is associated with longer hospital LOS and higher Clavien–Dindo grade.
Canales 2022 [24]	Identify whether POCUS measurements of the quadriceps and rectus femoris muscles can be used to discriminate between frail and not-frail patients and predict postoperative outcomes.	Musculoskeletal	Quadriceps depth, rectus femoris cross-sectional area and circumference	Preoperative ultrasound measurement of quadriceps depth shows promise in discriminating between frail and not-frail patients and is associated with unplanned SNF admission and post-op delirium.
Cozza 2021 [28]	Assess the feasibility of gastric POCUS in patients undergoing emergency abdominal surgery to predict the risk of post operative nausea and vomiting using gastroesophageal reflux disease-related parameters. Match the quantitative and qualitative measurements of gastric antrum to the clinical status, GI function, and actual postoperative course of patients, retrospectively.	Gastrointestinal (gastric)	Gastric volume	Sensitivity of gastric ultrasound varies depending on surgical technique. A dilated gastric antrum is significantly related to postoperative adverse outcomes and a careful ultrasound follow-up might help tailor postoperative nutrition and antiemetic therapy. In patients who experienced adverse events, antral cross-sectional area showed an average increase of more than 50% over a period of 72 h after surgery.
Cutright 2011 [29]	Determine if use of an ultrasound bladder-scanning device reduced the number of urinary catheters inserted in a med-surg unit.	Genitourinary	Bladder volume	Badder scanning for patients meeting med-surg unit criteria for “unable to void” resulted in significantly fewer catheterization compared to clinical criteria only.
Ramsingh 2021 [36]	Demonstrate the impact of applying a validated POCUS protocol in the post-anesthesia care unit (PACU) for patients with hypoxic and/or hypotensive events versus traditional bedside examinations.	Cardiac and pulmonary	Cardiac and pulmonary POCUS exams	Application of POCUS in PACU for hypotensive/hypoxic patients is associated with reduced PACU LOS and a reduction in number of suspected diagnoses.
NO IMPACT ON CLINICAL OUTCOMES				
Chui 2023 [26]	Assess the impact of POCUS lung and cardiac exam as part of preoperative assessment for hip fracture patients.	Cardiac and pulmonary	Lung and cardiac ultrasound (LUCAS)	LUCAS scans did not significantly change anesthetic plans, but they did provide reassuring information regarding severe cardiopulmonary conditions and supported recommendation of not delaying surgery for pending formal echocardiography. There were no changes in clinical outcomes because of preoperative LUCAS scan.
Gai 2018 [30]	Determine if presence of preoperative patent foramen ovale (PFO) as detected by cardiac POCUS is associated with post-op delirium in primary elective hip and knee arthroplasty. Determine the ease of performing bedside bubble study in perioperative setting, quality of US images, LOS, major CV and neurologic complications, and effects of anesthesia or analgesia techniques on delirium.	Cardiac	Presence of PFO	No conclusions could be drawn given the low incidence of PFO and post-op delirium and other major outcomes.
Haskins 2017 [32]	Determine the incidence of intra-abdominal fluid extravasation (IAFE) using focused assessment with sonography for trauma (FAST) exam after hip arthroscopy and whether presence of IAFE correlates with post-op pain and nausea/vomiting.	Gastrointestinal (non-gastric)	FAST exam for IAFE	Presence of IAFE correlated with greater increase in post-op pain from baseline, but there was no difference in post-op nausea/vomiting or LOS.
Ravetti 2023 [37]	Assess whether POCUS (lung, IVC, and cardiac) in the immediate postoperative period to guide hemodynamic management reduces incidence of AKI in high-risk surgery patients.	Vascular, pulmonary, and cardiac	Lung US pattern, IVC collapsibility, LV contractility (subjective)	Use of POCUS in the immediate postoperative period of high-risk surgery to guide hemodynamic management did not reduce incidence of AKI.
IMPACT ON ANESTHETIC PLAN OR MANAGEMENT				
Aissaoui 2022 [19]	Assess the ability of two point-of-care echocardiographic variables, velocity–time integral of the left ventricular outflow tract-passive leg raise (ΔVTE-PLR) and inferior vena cava collapsibility index (IVC-CI), to predict occurrence of post-induction hypotension (PIH)	Cardiac	ΔVTI-PLR and IVC-CI	ΔVTI-PLR, but not IVC-CI, could reliably predict the occurrence of PIH after general anesthesia.
Andruszkiewicz 2015 [20]	Evaluate the reliability of cardiac ultrasound performed by novice anesthesiologists during preoperative patient assessment. Evaluate the impact of these assessments on modification of patients’ management.	Cardiac	Cardiac function	Anesthesiologists with basic POCUS training can perform a reliable and accurate preoperative cardiac POCUS exam which had a significant impact on modification of anesthetic perioperative management.
Cowie 2011 [27]	Assess the indications, impact on clinical management, and accuracy of focused cardiovascular ultrasound performed by anesthesiologists in the perioperative period.	Cardiac	Cardiac function	When indicated, focused TTE alters peri-operative management of most patients and major clinical findings on focused TTE correlate with formal TTE in 92% of cases.
Szabo 2023 [39]	Assess whether preoperative US-based protocol for fluid administration can prevent postinduction and early intraoperative hypotension. Secondary aim was impact of protocol postoperative lactate level, urine output, and lung ultrasound score.	Vascular and pulmonary	IVC CI, beside lung ultrasound in emergency, LUS	Preoperative fluid administration based off a POCUS fluid replacement protocol can prevent early intraoperative hypotension and guide timing of fluid administration.
Van de Putte 2017 [40]	Evaluate the incidence of full stomach in a population of fasted patients presenting for elective surgery using gastric POCUS. Define the gastric volume distribution; association between gastric fullness, fasting intervals, and co-morbidities; anesthetic management changes; and incidence of aspiration.	Gastrointestinal (gastric)	Gastric fullness	Some patients may present with a full stomach despite recommended fasting for elective surgery; this finding changed their anesthetic management. There were no aspiration events to correlate with gastric fullness.
IMPACT ON DIAGNOSTIC ACCURACY				
Basumatary 2023 [21]	Determine incidence of pulmonary congestion diagnosed by lung US in patients with varied fluid administration.	Pulmonary	B line quantity and quality	Duration of surgery, large-volume intraoperative fluid administration, and net fluid balance lead to perioperative lung congestion. Lung US can detect lung congestion before clinical signs and symptoms.
Cavallari 2015 [25]	Evaluate the percentage of satisfactory diagnosis of handheld echocardiography (HHE) compared to standard TTE in preoperative patients. Evaluate the mean duration and wait time to perform both exams.	Cardiac	Cardiac function	There was no difference in percentage of satisfactory diagnosis between HHE and standard TTE. HHE was faster to perform and had shorter wait time to examination.
Szabó 2021 [38]	Identify characteristics with the potential of recognizing patients at risk for postoperative pulmonary complications (PPCs) by comparing LUS of patients with and without PPC in a 24 h postop timeframe.	Pulmonary	LUS	Persistently high postoperative LUS at 24 h identify patients at risk of or in an early phase of PPCs.
Wu 2023 [41]	Assess the impact of perioperative variables on atelectasis and lung aeration using lung ultrasound and their correlation with postoperative oxygenation.	Pulmonary	LUS	Patients with high LUS had higher BMI, lower post-op PaO_2_, and were more likely to be in lateral decubitus position (compared to supine). Age and LUS in PACU were independently associated with hypoxemia. Lung POCUS can help with early detection of perioperative atelectasis and lung aeration in the early post-op period.
ASSOCIATION OF US PARAMETER WITH CLINICAL VARIABLE				
Beaubien-Souligny 2018 [22]	Assess whether the renal resistance index (RI) predicted an increase in cardiac output (CO) following PLR in patients admitted to the intensive care unit after cardiac surgery.	Renal	Renal artery RI	Decrease in RI was associated with increase in CO following PLR. No association was found between change in RI following fluid bolus and increase in CO.
Goel 2020 [31]	Evaluate the impact of sonographically detected perioperative atelectasis on the need for postoperative oxygen supplementation, bronchodilator therapy, and assisted chest physiotherapy.	Pulmonary	Lung ultrasound score (LUS)	Higher LUS correlated with prolonged weaning time and change in score correlated with change in PaO_2_/FiO_2_ ratio.
Kaydu 2019 [33]	Define the relationship between IVC measurements and blood urea nitrogen (BUN)/creatinine (Cr) ratio in preoperative patients.	Vascular	IVC CI, inspiratory and expiratory diameter	No relationship was found between bedside measurement of IVC parameters and BUN/Cr ratio to predict preoperative dehydration.
Lamm 2022 [34]	Evaluate whether gastric volume (full vs. empty) on post-op day one correlates with measures of delayed bowel function after colorectal surgery.	Gastrointestinal (gastric)	Gastric volume	Patient identified as having full stomachs took longer to achieve GI-3 recovery (tolerating a regular diet with either flatus or a bowel movement).
Pettey 2022 [35]	Describe the relationship between the hepatic venous flow patterns and development of acute kidney injury (AKI) after cardiac surgery.	Vascular	Pulse-wave doppler of hepatic venous flow	Hepatic vein flow wave ratio (S wave to D wave) is associated with development of AKI, although they were not predictive of AKI development in multivariate regression models.
Yamanaka 2022 [42]	Evaluate the accuracy of renal artery pulsatility index (RAPI) in early detection of AKI after digestive surgery.	Renal	RAPI via renal interlobular artery velocities	Preoperative, POD4, and POD7 RAPI with cutoff > 1.6 is associated with perioperative AKI. RAPI is useful for early detection of AKI after digestive surgery.

## Supplementary Materials

The following supporting information can be downloaded at: https://www.mdpi.com/article/10.3390/jcm14207429/s1, Table S1: Systematic Review Search Strategies; Table S2: Quality and Bias Assessment Consensus Data; Table S3: PRISMA 2020 Checklist.

## Abbreviations

AKI, acute kidney injury; ASA, American Society of Anesthesiologists; POCUS, point-of-care ultrasound; RCT, randomized clinical trial; TTE, transthoracic echocardiogram
